# MENTAL AND PHYSICAL HEALTH OF OLDER ADULTS: INSIGHTS FROM CROSS-NATIONAL CONTEXTS

**DOI:** 10.1093/geroni/igae098.1662

**Published:** 2024-12-31

**Authors:** Jennifer Ailshire, Eunyoung Choi, Emma Nichols

**Affiliations:** Chair; Co-Chair; Discussant

## Abstract

Comparing the aging experience across country contexts offers a unique opportunity to determine whether observed differences in mental and physical health of older adults reflect a universal aging process or if they depend on the unique social and economic contexts in which older adults live. The HRS-family of surveys consists of nationally-representative, longitudinal studies of aging conducted in many countries around the world that have data and measures that have been harmonized within the Gateway to Global Aging data platform. This resource provides remarkable opportunities for cross-national comparative analyses of social and economic determinants of health and well-being among older adults. The papers in this session use these harmonized data to compare the aging experience across different social and economic exposures in multiple countries from around the world, including examinations of: 1) the role of exposure to recession and workplace security on psychological stress of older adults in the US and Korea; 2) the importance of resources and social status in mental health among widowed older adults in the US and India; 3) how caregiving relates to depression among informal family caregivers across 19 countries; and 4) the importance of migration status and country of residence for multimorbidity among Mexican Older adults in the US and Mexico. This symposium provides a comprehensive examination of how workplace, family, caregiving, and migration experiences contribute to health and well-being among older adults around the world.

